# Cost-effectiveness of cognitive behaviour therapy versus talking and usual care for depressed older people in primary care

**DOI:** 10.1186/1472-6963-11-33

**Published:** 2011-02-11

**Authors:** Amanda J Holman, Marc A Serfaty, Baptiste E Leurent, Michael B King

**Affiliations:** 1 Research Department of Mental Health Sciences, University College Medical School, London, UK; 2 The Priory Hospital North London, London, UK

## Abstract

**Background:**

Whilst evidence suggests cognitive behaviour therapy (CBT) may be effective for depressed older people in a primary care setting, few studies have examined its cost-effectiveness. The aim of this study was to compare the cost-effectiveness of cognitive behaviour therapy (CBT), a talking control (TC) and treatment as usual (TAU), delivered in a primary care setting, for older people with depression.

**Methods:**

Cost data generated from a single blind randomised controlled trial of 204 people aged 65 years or more were offered only Treatment as Usual, or TAU plus up to twelve sessions of CBT or a talking control is presented. The Beck Depression Inventory II (BDI-II) was the main outcome measure for depression. Direct treatment costs were compared with reductions in depression scores. Cost-effectiveness analysis was conducted using non-parametric bootstrapping. The primary analysis focussed on the cost-effectiveness of CBT compared with TAU at 10 months follow up.

**Results:**

Complete cost data were available for 198 patients at 4 and 10 month follow up. There were no significant differences between groups in baseline costs. The majority of health service contacts at follow up were made with general practitioners. Fewer contacts with mental health services were recorded in patients allocated to CBT, though these differences were not significant. Overall total per patient costs (including intervention costs) were significantly higher in the CBT group compared with the TAU group at 10 month follow up (difference £427, 95% CI: £56 - £787, p < 0.001). Reductions in BDI-II scores were significantly greater in the CBT group (difference 3.6 points, 95% CI: 0.7-6.5 points, p = 0.018). CBT is associated with an incremental cost of £120 per additional point reduction in BDI score and a 90% probability of being considered cost-effective if purchasers are willing to pay up to £270 per point reduction in the BDI-II score.

**Conclusions:**

CBT is significantly more costly than TAU alone or TAU plus TC, but more clinically effective. Based on current estimates, CBT is likely to be recommended as a cost-effective treatment option for this patient group if the value placed on a unit reduction in BDI-II is greater than £115.

**Trial Registration:**

isrctn.org Identifier: ISRCTN18271323

## Background

Depression in older people is common, frequently missed and undertreated [1]. Within the community it often becomes a chronic disorder with up to 74% of people remaining depressed one year after detection [2]. Depression is associated with an increase in the use of hospital and outpatient medical services [3-5], even after adjustments have been made for demographic, socioeconomic and functional status, and co-morbidity [5-7] Depressed patients are also more likely to end up in nursing homes [8]. All of this places an increased demand on health and social care resources [9] of which older people are traditionally high users.

Cognitive Behaviour Therapy (CBT) is a clinically effective and recommended treatment for depressive disorder in adults of all ages and is associated with continued improvement over time [10]. Older people in particular tend to be high utilisers of medical services and service demands increase with clinical depression [7,11]. CBT is effective for depressed older people in a primary care setting [12]. However, few studies have assessed whether psychological interventions such as CBT are cost effective for depressed older people treated in a primary care setting.

Bower et al (2000) conducted an analysis of the cost-effectiveness of CBT for depression in people of all ages, comparing CBT with non-directive counselling and usual general practitioner care [13]. Both non-directive counselling and CBT were associated with a reduction in depressive symptoms at four months, but this was not sustained through till 12 months follow up. There were no significant differences in direct costs (drugs, outpatient services, inpatient services, protocol therapy, travel costs), production losses, or societal costs between the three treatments at either four or 12 months. However, closer analysis of the data showed patients receiving usual general practitioner care alone recorded more GP consultations, greater use of antidepressants, and more psychiatric referrals. Unützer et al (2000) randomised 1,801 depressed primary care patients aged over 60 to usual care by their primary care doctor or collaborative care. The cost of the intervention was about £370 ($670; €550) per patient over 12 months which was substantially less than a year's worth of anti-dementia drugs (approximately £1,000) [14].

We present a cost-effectiveness analysis based on a randomised controlled trial of three treatments, Treatment as Usual (TAU), TAU plus CBT or TAU plus a talking control, for older people with depression presenting in primary care [12]. This design was used to balance known and unknown factors which may predict outcome and aimed to take into account the effects of "non-specific" factors in therapy, including face-to-face contact with another person, warmth and empathy. For the purpose of this paper we will refer to the three interventions as TAU, CBT or TC, while acknowledging the latter two were provided in addition to TAU.

### Aims

#### Health Economics Objective

First, to compare the average costs of care associated with CBT, a Talking Control (TC) or Treatment as Usual (TAU) for older people with depression. Secondly, to estimate the cost-effectiveness of CBT plus TAU compared with TAU alone for older people with depression using the change in Beck Depression Inventory-II (BDI-II) score at the trial end point. The analysis was conducted from the perspective of the UK Departments of Health and Social Services.

## Methods

The study was conducted in a primary care setting in North London between April 2004 and September 2007. The inclusion criteria were (1) a primary diagnosis of depressive disorder obtained from the Geriatric Mental State and History and Etiology Schedule [15]. (2) a score of 14 or higher on the BDI-II [16] (3) sufficient command of English to use CBT techniques; and (4) if taking an antidepressant, a stable dose of medication for at least 8 weeks prior to randomization. Informed consent was obtained from all participants. Full details on the design and methods employed in the clinical trial, which adhered to the CONSORT guidelines, have been reported in Serfaty et al, (2009) [12].

### The intervention

Patients allocated to CBT or TC were offered up to twelve sessions with a therapist accredited by the British Association of Behavioural and Cognitive Psychotherapists. Within the constraints of the number of sessions available, the patient and the therapist collaboratively agree when therapy should terminate. The TC was used to control for the main non-specific effects of therapy, and is most comparable to a befriending service. All patients received usual care as managed by their general practitioner. The project was approved by the ethics committee of Camden and Islington Community Health Services Trust and supported by the North Central London Research Consortium, LREC reference 03/37.

### Health service utilisation

Data on health service use was accessed via general practice medical records and collected using a modified version of the Client Service Receipt Inventory (CSRI) [17]. Data were collected for three month periods prior to all follow up points: baseline, 4 month post baseline and 10 month post baseline.

Direct treatment costs associated with the intervention, as well as community health service costs were included. Community health care included contacts with GP's, practice and district nurses, health visitors, psychiatrists, clinical psychologists, occupational therapists, physiotherapists, community psychiatric nurses and general counsellors. All prescribed medication was recorded. Data specifically for antidepressant medication is provided in detail in Serfaty et al (2009) [12]. No significant differences at baseline or during the course of the trial were observed and imipramine dose equivalents were similar in all 3 groups. Data relating to collecting of prescriptions or medication compliance were not available. However, as antidepressant prescribing was similar across groups, it is likely that the costs of medication were balanced.

Although it would have been helpful to obtain information on indirect costs such as production losses, patient time, and caregiver time and burden, this was not possible for ethical reasons. The bulk of the CSRI was collected through consultation with GP records. Inclusion of other costs would have entailed further data collection from a vulnerable group of patients already burdened with having to complete a number of rating scales.

The authors believed there was no reason why CBT should affect the use of acute hospital care services, and moreover, there was concern that any underlying differences in functionality between patient groups may result in differences in hospital utilisation, and therefore introduce unnecessary variance to the costs. While there was no rationale for including hospital utilisation in the analysis, these data were available and provided an opportunity to assess the reasons for admission and whether the decision to exclude these costs was appropriate.

### Unit costs

Costs are presented from the perspective of the UK Departments of Health and Social Services, in 2008 values (Table 1). Patient-level costs were calculated by multiplying frequency of contacts with health service providers with unit costs from *Unit Costs of Health and Social Care 2008 *[18]. Intervention costs were based on the number of sessions attended by each patient. Data for the number of therapy sessions was continuous and normally distributed and therefore tested using an unpaired t-test. There was no significant difference between the mean number of sessions attended for CBT (mean [SD], 7.09 [4.41] sessions) or TC (mean [SD], 7.58 [4.56] sessions) (p = 0.52). Total costs are presented as a cumulative total of health service costs and intervention costs.

**Table 1:** Unit Costs (£ 2008)

Community Health Services	Unit Cost	Reference (PSSRU Unit Costs 2008)
GP contact	36	Per clinic consultation lasting 11.7 minutes
Phoned GP for advice	22	phone call 7.1 mins
GP home visits	58	home visit 23.4 mins
Practice nurse	11	Per consultation
Phoned practice nurse	6.72	data not available. set as a proportion according to GP time consult: phone visit
District nurse	26	Per home visit
Health Visitor	16.5	£33 per hour inc quals; Assume 30 mins session.
Psychiatrist	106	Per contract hour
Clinical psychologist	41	Per professional chargeable hour.
Occupational Therapist	66	Average cost for a one to one contact.
Physiotherapist	42	Average cost for a one to one contact.
Community Psychiatric nurse	32	Nurse specialist (community). Per hour (including qualifications)
Counsellor/Indiv Therapist	40	Per hour of client contact. Counselling services in primary medical care.
**Intervention costs**		
CBT session	66	CBT session
Talking control session	24	Social worker assistant

### Statistical Analysis

Although detailed resource use data were collected, the sample size was calculated on the basis of expected clinical outcomes and not on the cost analysis and reported in Serfaty et al [12]. The cost analysis was based on patients with complete cost-data only, given very little was missing (n = 6/204, 3%). As costs were not normally distributed, analysis of variance (ANOVA) was performed on log-transformed cost data. As data had only been collected for three months prior to each follow up period (at 4 and 10 months), the 'gaps' were estimated as a proportion of the costs incurred during the subsequent follow up periods. The primary analysis was of total costs at the trial end point (10 months), but we also report costs at 4 months follow up. A sub-analysis was conducted for mental health services using a chi-squared test (including visits to psychiatrists, clinical psychologists, occupational therapists, community psychiatric nurse and counsellors). Around 90% of patients did not use any mental health services, therefore neither a t-test, nor a non-parametric test such as Kruskall Wallis, could be used on the continuous variable "number of contacts", hence the variable was dichotomised. Baseline costs were examined for differences between groups, but were not used to adjust the results.

Effectiveness was defined as the change in BDI-II from baseline to follow up. As there were no significant differences between groups on health-related quality of life, we did not perform a cost-effectiveness analysis using this outcome measure. Multiply-imputed values generated for the effectiveness analysis (Serfaty et al., 2009) were used in the case of missing BDI-II follow-up values. Outcomes data were missing for 13% and 18% of the patients at 4 and 10 months respectively, with no difference across arms. We based our analysis on the imputed outcomes data used for the effectiveness trial [12]. Outcomes were imputed using multiple imputation [19], using the ice command in STATA to generate 5 sets of imputed values.

### Cost-effectiveness analysis

We performed an incremental cost-effectiveness analysis (CEA) comparing CBT with both TC and TAU, calculating the incremental cost-effectiveness ratios (ICER), i.e. the difference in average costs divided by the difference in average effects between groups. In order to capture the uncertainty around the estimates, 1000 nonparametric bootstrap replications were generated from the sets of multiply imputed data, and mean cost and effect were plotted in a cost effectiveness plane.

As we do not know the threshold willingness to pay value for additional effectiveness associated with CBT, the probability that CBT will be considered cost effective was calculated for a range of threshold values and presented in a cost effectiveness acceptability curve (CEAC).

Data were analysed and prepared in Stata Version 11 (Stata Corp, College Station, Tex.), and bootstrapping and cost-effectiveness analysis was undertaken in MS Excel.

Our a priori hypothesis aimed to compare costs in relation to our main outcome measure, the BDI-II. Serfaty et al (2009) did not find any significant difference in Euroqol scores between groups at any time point (change in EQ-5D from baseline to follow -up: 0.04 CBT; 0.06 TAU) [12]. A cost-effectiveness analysis on our subsidiary outcome measure would therefore not be appropriate.

## Results

### Recruitment and follow through

Of the 204 participants who entered the study, 83 (40.7%) self referred, 72 (35.3%) were referred by their GP and 49 (24.0%) were recruited through GP data bases. The majority were female (n = 162; 79.4%), white (n = 154; 75.5%) and aged 74.1(SD 7.0) years. One hundred and fifty (73.5%) were antidepressant free at baseline. There were no differences in any of the above characteristics in those allocated Treatment as Usual (TAU), TAU plus CBT (n = 70), or TAU plus a Talking Control (n = 67). The availability of cost data was high. Among 204 patients recruited into the trial, 198 patients had complete cost data at 10 months follow up (post baseline). Six cases were missing having all withdrawn from the trial after baseline.

### Health service utilisation

The majority of community health service contacts were made with general practitioners (85%). Overall, the average number of contacts were very similar between CBT and TAU groups, and lowest in the TC group (Table 2). Significant differences are tested below in the section on average costs, where resource counts are multiplied by unit costs.

**Table 2:** Average number of contacts with community health services (over both follow up periods)

	CBT n = 67	TC n = 65	TAU n = 66	Total n = 198
GP contacts	6.97	6.08	7.17	6.74
Phoned GP for advice	0.28	0.49	0.59	0.45
GP home visits	2.91	1.35	2.14	2.14
Practice nurse	3.10	1.92	2.95	2.67
Phoned practice nurse	0.10	0.22	0.05	0.12
District nurse	2.88	0.40	2.92	2.08
Health Visitor	0.00	0.00	0.00	0.00
Physiotherapist	0.55	0.72	1.11	0.79

### Mental health service use

Patients in the CBT group generally reported fewer contacts with mental health services than patients in other groups (Table 3). Patients in the TAU group recorded the highest average number of contacts. However, there were no statistically significant differences in the proportion of patients who had at least one contact with mental health services at either follow up point (four months post-baseline: CBT 8.96%, TC 7.69%, TAU 16.67% p = 0.207; 10 months post-baseline CBT 11.94%, TC 12.31%, TAU 13.64% p = 0.953). As the numbers of patients accessing mental health services was very small (ten or less in each group), it may not have been possible to detect meaningful differences between the treatment groups.

**Table 3:** Average number of contacts with mental health services (over both follow up periods)

	**CBT**	**TC**	**TAU**	**Total**
***Mental health services:***				
Psychiatrist	0.09	0.09	0.11	0.10
Clinical psychologist	0.21	0.60	0.35	0.38
Occupational Therapist	0.03	0.05	0.18	0.09
Community Psychiatric nurse	0.00	0.09	0.08	0.06
Counsellor/Therapist	0.27	0.00	0.47	0.25

### Hospitalisation and reasons for admission

The reasons for hospital admission were varied, although the majority were unplanned emergency admissions that were unlikely to have been influenced by the psychological interventions (e.g. orthopaedic surgery). Only one hospital admission was related to depression. Outpatient appointments were also unrelated to depression. The number of inpatient admissions did not differ across arms, or over time (overall proportion of admissions: baseline 12%, four months: 10%, 10 months: 13%). These findings supported the decision to exclude hospital costs from the analysis as they would have only introduced a source of unwanted variation to the costs data.

### Average Costs

Mean per patient costs are shown in Table 4 by treatment group. Histograms showed cost data was highly skewed, therefore tests of significance using one way ANOVAs were performed on log-transformed data. Results showed no significant difference between groups in baseline costs (p = 0.0703). No difference in community health service costs were shown at either four or 10 months follow up. However, total per patient costs (including intervention costs) were significantly higher in the CBT group compared with the TAU group at both four months (difference £380, 95% CI: £254, £311, p < 0.001) and 10 months follow up (difference £427, 95% CI: £56, £787, p < 0.001). Overall, total per patient costs were lowest in the TC group.

**Table 4:** Mean per patient costs (£) by group (SD)

	CBT n = 67	TC n = 65	TAU n = 66	p value
Baseline Costs (3 months)	180 (127)	163 (125)	225 (193)	0.0703
Intervention costs	437 (276)	180 (102)	-	0.0000
Community health service costs at 4 months	304 (312)	240 (199)	361 (340)	0.1604
Community health service costs at 10 months	1027 (1118)	704 (523)	1037 (1005)	0.0733
Total costs at 4 months	741 (437)	419 (206)	361 (340)	0.0000
Total costs at 10 months	1464 (1198)	884 (537)	1037 (1005)	0.0001

* costs cannot be equated to the average numbers of contacts reported above, as they have been extrapolated to estimate the gaps in between follow up periods.

**Figure 1:**
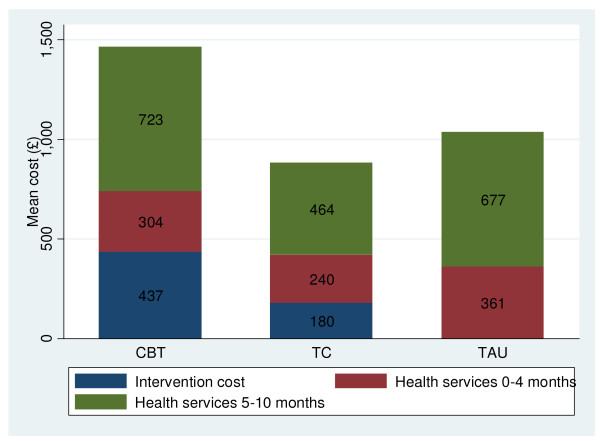
**Average costs, by treatment group**.

Figure 1 demonstrates how there is very little difference between groups in community health service costs. The high total cost of CBT is being driven by the cost of the intervention (£437, in comparison with £0 for TAU), and a relatively higher spend on community health services (£1027, in comparison with £704 for TC). CBT patients may be encouraged to seek help for their physical health problems, which incurs increased health contacts.

### Effectiveness

Effectiveness was assessed using the Beck Depression Inventory-II. Intent to treat (ITT) analysis found a -3.07 (95% CI, -5.73, -0.42) and -3.65 (-6.18, -1.12) improvement in BDI-II score in favour of CBT against TAU and TC respectively [12]. These findings were confirmed with complier's average causal effect (CACE) analysis, adjusted for BDI-II, which showed the benefit of CBT as 0.40 of a BDI-II point (95% CI: 0.01-0.72) per therapy session. Reductions in BDI-II scores were significantly greater in the CBT group at four months and 10 months follow up compared with both the TAU group and the TC groups (Table 5). Twenty two (11%) and 31 (16%) patients did not complete the 4 and 10 months BDI-II questionnaire, respectively, and their outcome was imputed by multiple imputation. Euroqol data showed no significant differences by intervention group, with a 0.05 points (95% CI, -0.04, 0.14) higher increase at 4 months for CBT compared to TAU, and 0.04 (95% CI, -0.05, 0.12) for CBT against TC [12].

**Table 5:** Mean change in BDI-II score from baseline

Clinical Effectiveness	CBT	TAU	TC	CBT vs TAU	CBT vs TC
Reduction in BDI-II score 4 months	9.4	6.6	5.8	2.8	3.6
95% CI	7, 11	4, 9	4, 8	-0.4, 6.0	0.6, 6.4
P-value				0.084	0.010
Reduction in BDI-II score 10 months	9.7	6.2	6.0	3.6	3.5
95% CI	8, 12	4, 8	4, 9	0.7, 6.5	0.3, 6.5
P-value				0.018	0.020

### Cost-effectiveness

**Figure 2:**
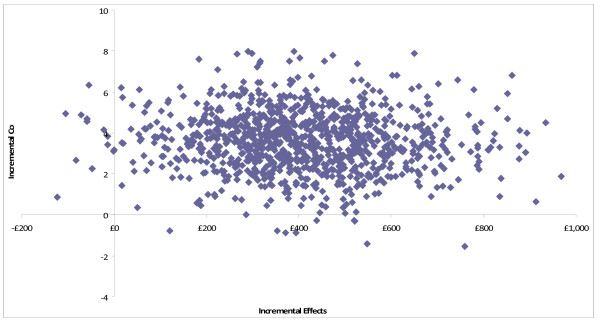
**Incremental cost-effectiveness plane for CBT vs TAU at 10 months follow up. Costs are direct costs including treatment in GBP**. Effects are the reduction in BDI-II score from baseline to 10 months follow up. Cost and effect pairs were estimated with 1000 bootstrap replications.

**Figure 3:**
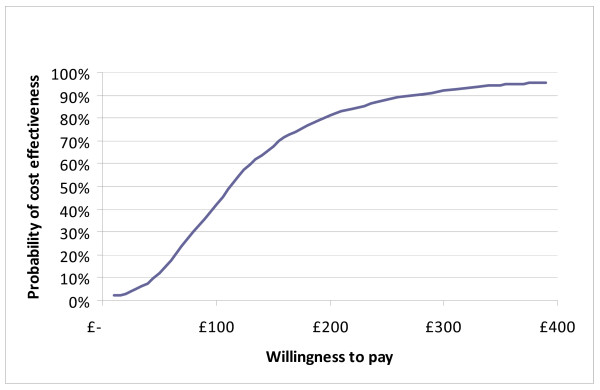
**Cost-effectiveness acceptability curve for CBT vs TAU at 10 months follow up**.

At both four and 10 months follow up, CBT was more costly, and more effective than TAU and TC. Dividing these incremental costs by the incremental effectiveness allows us to estimate the incremental cost-effectiveness ratio (ICER) for CBT compared to TAU (Table 6) and TC (Table 7), at both follow up points. Our primary analysis was the cost-effectiveness of CBT compared with TAU at trial end point, being 10 months. In this case, CBT was associated with an ICER of £120 per additional point reduction in BDI score. Uncertainty in the ICER was estimated through bootstrapping, and by plotting the 1000 replications of mean cost and effect differences on a cost-effectiveness plane (Figure 2). As 98% of re-samples fell within the top right-hand quadrant of the plane, this indicates a high likelihood of CBT plus TAU having higher costs and better outcomes as measured by the BDI-II. Whether this incremental improvement in outcome represents good value for money given the likelihood of higher service costs is a value judgement. However, this judgement can be informed by constructing CEACs, which show the likelihood that adding CBT to TAU is more cost-effective than TAU alone for different values placed on a unit improvement in the BDI-II. The CEAC shows a 90% probability that CBT will be considered cost effective if purchasers are willing to pay up to £270 per point reduction in BDI-II score (Figure 3). CBT has a greater than 50% likelihood of being the most cost-effective option only if the value placed on a unit reduction in BDI-II score is above £115.

**Table 6:** Cost-effectiveness of CBT compared to TAU at 4 and 10 months follow up

4 months follow up	Mean	95% CI
Incremental cost	£380	£254, £511
Incremental effectiveness (average point reduction in BDI)	2.8	-0.4, 6.0
Incremental cost-effectiveness (per point reduction in BDI score)	£133	
**10 months follow up**		

Incremental cost	£427	£56, £787
Incremental effectiveness (average point reduction in BDI)	3.6	0.7, 6.5
Incremental cost-effectiveness (per point reduction in BDI score)	£120	

**Table 7:** Cost-effectiveness of CBT compared to TC at 4 and 10 months follow up

4 months follow up	Mean	95% CI
Incremental cost	£322	£212, £448
Incremental effectiveness (average point reduction in BDI)	3.6	0.7, 6.5
Incremental cost-effectiveness (per point reduction in BDI score)	£89	
**10 months follow up**		

Incremental cost	£580	£280, £930
Incremental effectiveness (average point reduction in BDI)	3.5	0.3, 6.5
Incremental cost-effectiveness (per point reduction in BDI score)	£167	

## Discussion

CBT is significantly more costly and more effective than TAU, and TAU plus a talking control. As discussed in Serfaty et al (2009), the widely perceived benefits of 'talking' about problems were not translated into reductions in depression scores. These results confirm that CBT is an effective psychological intervention, but it comes at a cost. The additional costs of CBT relative to treatment as usual (£380 at four months; £427 at 10 months) were driven by the cost of the intervention. Whilst there was no significant difference in community health service costs at either follow up point, patients in the CBT group did record fewer contacts with mental health services than patients in the other groups, suggesting CBT has a substitute effect for other mental health services.

Our primary analysis concerned the cost-effectiveness of CBT compared with TAU at 10 months follow up. Results showed CBT was associated with an ICER of £120 per point reduction in BDI-II score compared to TAU. Whether or not CBT is cost effective and represents value for money depends on how much purchasers are willing to pay for an additional point reduction in BDI-II score. We estimated a 90% probability that CBT would be considered cost effective if purchasers are willing to pay up to £270 per point reduction in BDI-II score, and a 50% likelihood of being the most cost-effective option only if the value placed on a unit reduction in BDI-II score is above £115. These threshold analyses aim to indicate an upper confidence limit for cost effectiveness, meaning there is a high probability that CBT would cost no more than an additional £270 per point reduction in BDI score. However, it is more likely the extra cost of CBT would be closer to the mean value of £120 per point reduction in BDI score.

Few studies have been published on the cost-effectiveness of CBT for older people in primary care. However, our results are comparable to those published by Bower et al (2000) who reported no significant difference between groups in health service costs, and also observed more referrals to mental health services among those receiving GP care only [13].

These results are limited by our ability to interpret cost-effectiveness associated with changes on a depression scale, and this is an area in which more information is needed. For example, future research may focus on conducting a willingness to pay study among potential purchasers. However, interpreting and comparing ICER's would also be made easier by expressing results in quality adjusted life years (QALYs) using a measure such as the Euroqol. While Euroqol data was collected in this trial, results showed no significant difference between groups. Although the Euroqol is often used to determine cost-effectiveness, and may be useful in other depressed populations [20,21], in older people it may be too generic and lack the sensitivity required to detect meaningful differences between groups. Alternatively, CBT may be effective for targeting depression, but not improving quality of life in older people. Indeed, these findings are consistent with feedback from our therapists who reported that patients did not necessarily engage in increased activity because of associated physical problems, but their perspective about the problem changed. In either case, these findings would suggest that the BDI-II may be more appropriate than the EQ-5D for evaluating the economic benefits of treatment in this patient group.

We acknowledge that a limitation of this study is the possibility that patient-level costs may have been influenced by need-related factors, such as disease severity, functionality and deprivation. In future studies a measure of dependency should be included, so that results can be adjusted for any imbalances between groups. Both the Barthel index [22], and the ECOG performance status measure for cancer [23] are commonly used.

This cost analysis points to the importance of a rationale for the inclusion of certain costs in order to reduce unwanted variability in cost data. Our analysis showed inpatient admissions did not appear to be influenced by the intervention, and were therefore excluded. Inclusion of all possible health service costs in order to provide a comprehensive picture of the full costs of care may actually mask important differences in health service use that occur in only a few areas, by adding increased 'noise' around costs, which are already highly variable. Advice suggests only costs likely to differ between treatment groups should be collected, as economic analysis is primarily concerned with marginal costs [24]. Collection of resource use data can also be very expensive and the cost of this extra information may not be justified if only costs directly associated with providing the intervention are expected to differ between groups.

## Conclusions

CBT is more likely to be cost effective compared with treatment as usual for older people presenting with depression in primary care, assuming a willingness to pay threshold of more than £115 per point reduction in BDI-II score. The cost-effectiveness of CBT in this patient group depends on how much purchasers are willing to pay for reductions in depression scores. Future economic evaluations of psychological interventions studies should focus on marginal costs that are expected to vary between groups as a result of an intervention and also differentiate between contact with mental health and other services.

## Competing interests

The authors declare that they have no competing interests.

## Authors' contributions

AH designed and carried out the cost-effectiveness analysis and drafted the manuscript. BL was responsible for data extraction, cleaning, manipulation and analysis. MS conceived the main clinical trial and helped draft the manuscript. MK provided guidance on the study design. All authors read, edited and approved the final manuscript.

## Pre-publication history

The pre-publication history for this paper can be accessed here:

http://www.biomedcentral.com/1472-6963/11/33/prepub
